# Lysophosphatidylcholine acyltransferase 2 (LPCAT2) co-localises with TLR4 and regulates macrophage inflammatory gene expression in response to LPS

**DOI:** 10.1038/s41598-020-67000-x

**Published:** 2020-06-25

**Authors:** W. Abate, H. Alrammah, M. Kiernan, A. J. Tonks, S. K. Jackson

**Affiliations:** 1 Biomedical Research Group, Faculty of Health, University of Plymouth, Derriford, Plymouth, PL6 8BU UK; 2 School of Medicine, Cardiff University, Heath Park, Cardiff, CF14 4XN UK; 3 Present Address: University of Exeter Medical School, Truro, TR1 3HD UK

**Keywords:** Sepsis, Sepsis

## Abstract

Despite extensive investigations, an effective treatment for sepsis remains elusive and a better understanding of the inflammatory response to infection is required to identify potential new targets for therapy. In this study we have used RNAi technology to show, for the first time, that the inducible lysophosphatidylcholine acyltransferase 2 (LPCAT2) plays a key role in macrophage inflammatory gene expression in response to stimulation with bacterial ligands. Using siRNA- or shRNA-mediated knockdown, we demonstrate that, in contrast to the constitutive LPCAT1, LPCAT2 is required for macrophage cytokine gene expression and release in response to TLR4 and TLR2 ligand stimulation but not for TLR-independent stimuli. In addition, cells transfected to overexpress LPCAT2 exhibited increased expression of inflammatory genes in response to LPS and other bacterial ligands. Furthermore, we have used immunoprecipitation and Western blotting to show that in response to LPS, LPCAT2, but not LPCAT1, rapidly associates with TLR4 and translocates to membrane lipid raft domains. Our data thus suggest a novel mechanism for the regulation of inflammatory gene expression in response to bacterial stimuli and highlight LPCAT2 as a potential therapeutic target for development of anti-inflammatory and anti-sepsis therapies.

## Introduction

Sepsis is a life-threatening condition that is estimated to result in as many as 20,000 deaths per day Worldwide^1^. Despite extensive investigations and numerous clinical trials, there is currently no effective therapy for sepsis^2^. Thus, identification of new therapeutic options for patients suffering from sepsis is paramount. Bacterial molecules such as lipopolysaccharide (LPS) play key roles in the development of sepsis. Detection of LPS by innate immune cells is vital for host defense against Gram-negative bacteria yet unregulated responses can produce excessive cytokine release, which can ultimately lead to the cardiovascular derangements and organ dysfunction that are the hallmarks of severe sepsis^3–5^. LPS activates the TLR4/MD2 complex on cells such as macrophages leading to intracellular signalling and activation of NF-κB and MAPKs which culminates in the release of inflammatory cytokines^6^ and lipid mediators^7^. The approach of blocking individual inflammatory mediators has not proved successful in clinical sepsis trials^1,8^ and therefore a better understanding of the inflammatory pathways activated by TLRs is required to develop effective therapies.

In addition to being the major and fundamental structural components of biological membranes, phospholipids (PLs) and their metabolites play critical roles in cell signaling and activation^9^. PL metabolites, such as lysoPLs (LPL) and oxidised PL (oxPL), have been shown to mediate inflammatory responses and to influence outcome in experimental models of sepsis^10^. Diverse phospholipid composition profiles are required for membrane fluidity, vesicle trafficking, and special domain formation for signalling^11,12^. The mechanistic details of the remodelling process that underlie different phospholipid compositions have recently become better understood. Membrane diversity and asymmetry is maintained by the concerted deacylation and reacylation of phospholipids in the so-called ‘Land’s Cycle’^13^. Deacylation reactions are mediated by phospholipases, in particular phospholipase A2 (PLA2), the activity of which is increased in inflammation. The reacylation of the major membrane phospholipid, phosphatidylcholine (PC), is performed by a family of LysoPC acyltransferase (LPCAT) enzymes. Recent studies have revealed four enzymes with LPCAT activity, namely LPCAT1, LPCAT2, LPCAT3 and LPCAT4^14^. In addition to LPCAT activity, LPCAT1 and LPCAT2 have been shown to express lyso-platelet activating factor acetyltransferase (LPAFAT) activity^15,16^. LPCAT1 has been found to be highly expressed in lung tissue where it regulates surfactant lipid generation^15,17,18^. LPCAT2 has been reported to be expressed in inflammatory cells and to be up-regulated by LPS stimulation^15^.

Acyltransferase activity has been linked to inflammatory disease. For example the ratio of PC/LysoPC in plasma has been shown to be a measure of inflammation in rheumatoid arthritis^19^, and lysoPC, a major component of oxidized low density lipoprotein, has been shown to be beneficial in experimental sepsis models^20^. Plasma LysoPC levels are decreased in sepsis patients and decreased LysoPC/PC ratios are highly correlated with sepsis mortality^10^.

Our group has been investigating the role of phospholipid metabolites in the inflammatory responses to LPS. Our previous studies, using global pharmacological inhibition of LPCAT, suggested a key role for these enzymes in inflammation although the specific isoform of LPCAT responsible for these effects was not elucidated^11,21,22^. LPCAT1 and LPCAT2 have been shown to be differentially expressed and have distinct phospholipid metabolising functions [15,16], and therefore the identity of the LPCAT isoform regulating inflammation will be key to the development of targeted anti-inflammatory therapies. In the current study, we exploited the recent availability of antibodies and sequence data for LPCAT1 and 2^15,17,18^ to develop RNA interference (RNAi) technology to determine the role played by LPCAT1 and 2 in bacterial-induced inflammatory responses. We here show that LPS stimulates association between LPCAT2 and TLR4 and the translocation of LPCAT2 into membrane lipid raft domains. We also show that cells overexpressing LPCAT2 produce enhanced inflammatory gene expression in response to LPS and other bacterial ligands and that selective knockdown of LPCAT2 inhibits the inflammatory gene expression. Thus, our data support a major role for LPCAT2 in the regulation of inflammatory responses to LPS and other bacterial TLR ligands.

## Results

### LPCAT2 is expressed in resting macrophages and is induced by TLR4 and TLR2 ligands

**Figure 1:**
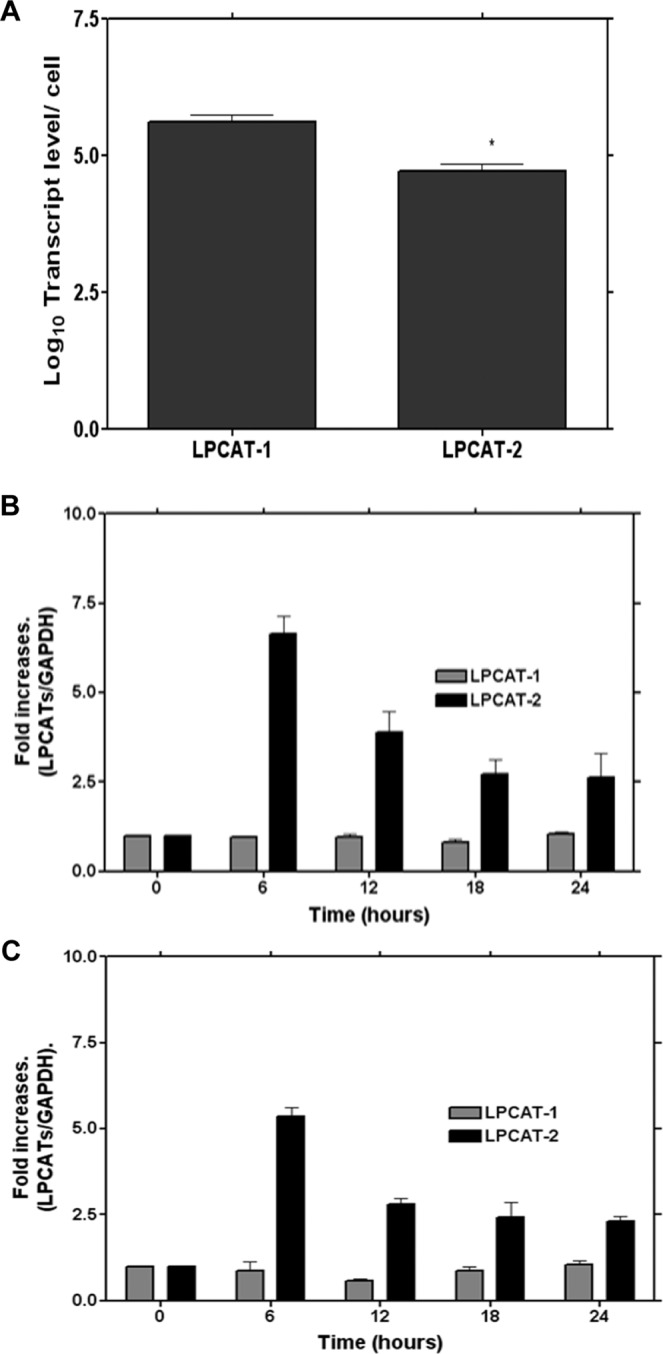
The level of expression of LPCAT1 and 2 and their induction by LPS or LTA in RAW264.7 cells. The copy number for the LPCATs in RAW264.7 cells were determined as described in the methods section. In resting cells the level of LPCAT1 transcripts was relatively higher than LPCAT2 (**A**). LPCAT 1 and LPCAT2 mRNA expression in RAW264.7 cells stimulated either LPS (1 µg/mL) (**B**) or LTA (10 µg/mL) showing the inducibility of LPCAT2 by these ligands (**C**). n = 3; *p < 0.05 Vs LPCAT1.

The presence and the absolute transcript level of LPCAT mRNA was determined using a real time quantitative PCR. RAW264.7 cells expressed both LPCAT1 and -2 in a resting state. The amount of LPCAT1 transcript was eight fold higher than LPCAT2 (5.575 + 0.15 Vs. 4.671 + 0.14, Log_10_ Mean transcript level + SE, p = 0.0115) in unstimulated cells (Fig. 1A). It has been shown that incubation of macrophages with TLR4 or -9 ligands for 16 hours up-regulates the mRNA expression of LPCAT2^15^. In the present work, we further examined the induction of both LPCAT1 and 2 by the TLR4 ligand LPS and the TLR2 ligand lipoteichoic acid (LTA). We found that the level of LPCAT2 expression was significantly up-regulated when the cells were stimulated with either TLR2 or TLR4 ligands after 6 hours. In contrast, LPCAT1 mRNA expression was not significantly altered when the cells were stimulated with LPS or LTA up to 24 hours compared to resting cells (Fig. 1B,C).

### LPCAT2 translocates to membrane lipid raft domains upon cell stimulation with LPS

**Figure 2:**
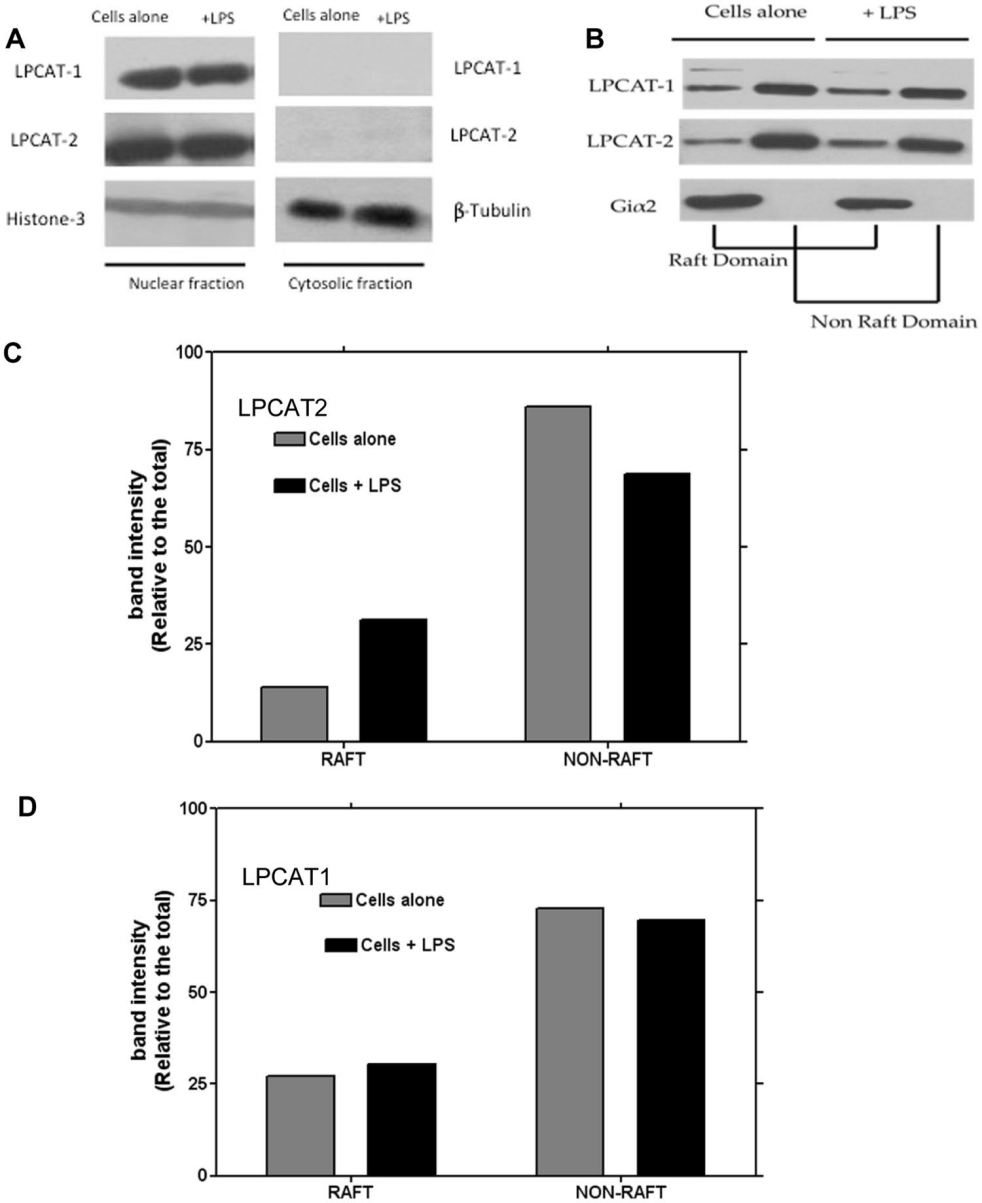
Sub-cellular location of LPCAT1 and LPCAT2. Different cellular fractions were isolated and each of the fractions was probed for LPCAT1 or 2 by immunoblotting. Both LPCAT1 and 2 exist in the nuclear- rich fractions (Histone H3 used as a marker) but not in the cytosol (β-tubulin used as cytosolic marker) and their relative abundance does not change following LPS stimulation (**A**). Isolation of membrane fractions showing that the majority of LPCAT1 and 2 is expressed in the non-raft membrane domain but the level of LPCAT2 is increased in lipid raft domains after LPS stimulation while the level of LPCAT1 remains unchanged (**B**). Densitometry analysis of the fractions from B are shown for LPCAT2 in (**C**) and LPCAT1 in (**D**).

To assess the localisation of LPCAT1 and 2 inside the cells, the cytosolic, nuclear and membrane fractions of resting cells and cells stimulated with LPS were isolated by successive detergent extraction^24^. The distribution profile of LPCAT1 and -2 inside the cells was identical and both enzymes were present in the membrane and nuclear factions but not in the cytosol (Fig. 2A). In resting cells, the majority of the LPCAT1 and LPCAT2 were found in the non-raft domain of the membrane (Fig. 2B). However, on stimulation of cells with LPS, LPCAT2 was found to translocate to the lipid raft domain in contrast to LPCAT1 which did not increase in the lipid raft fraction (Fig. 2B–D).

### Knockdown of LPCAT2 by siRNA down-regulates inflammatory cytokine gene expression

**Figure 3:**
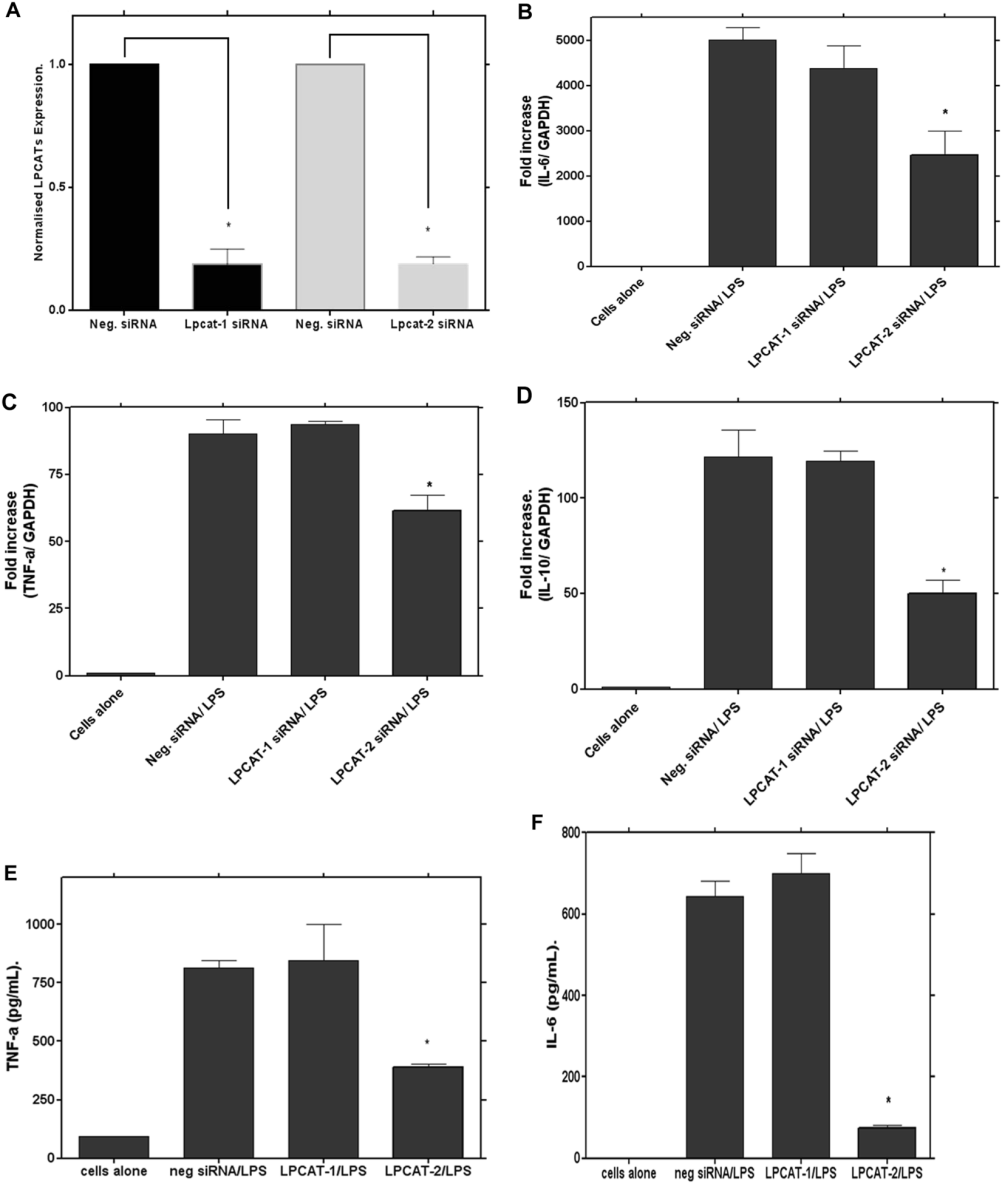
Inhibition of LPCAT2 by siRNA significantly down-regulates LPS-induced inflammatory cytokine gene expression and protein release. Treatment of RAW264.7 cells with LPCAT1 or LPCAT2 siRNA resulted in 81.35 + 6.4 and 80.09 + 1.36 per cent knockdown of LPCAT1 and 2 expression, respectively, compared to the treatment with a control negative siRNA (**A**). Following siRNA knockdown of LPCAT1 or LPCAT2, cells were stimulated with LPS at 1 µg/mL, for either 4 hours, for cytokine mRNA expression (**B–D**) or 24 hours for protein release (**E,F**). *p < 0.05 Vs negative siRNA (**B–F**).

The expression of LPCAT1 and 2 were successfully suppressed by small interfering RNA with 81.35 + 6.4% and 80.09 + 1.36 per cent knockdown respectively with no significant effect of cytotoxicity from the transfection reagent (Fig. 3A). Using this optimal siRNA knockdown of LPCAT1 and -2 expression, the effect of LPCAT1 or -2 silencing on LPS-induced cytokine gene expression was initially investigated using real time quantitative PCR. We here demonstrate, for the first time, that the inhibition of LPCAT2 expression significantly down-regulates the mRNA expression of LPS-induced TNF-α, IL-6 and IL-10 (Fig. 3B–D). The effect of LPCAT2 inhibition on LPS-induced protein release was also studied using a Milliplex MAP mouse multiplex kit. The release of LPS-mediated inflammatory cytokines was also markedly reduced following the siRNA suppression of LPCAT2 (Fig. 3E,F) which mirrored the reduction in mRNA of the inflammatory cytokines. In contrast to LPCAT2 silencing, siRNA knockdown of LPCAT1 had no effect on LPS-induced inflammatory cytokine gene expression at either mRNA or protein level (Fig. 3B–F).

The modulation of RAW264.7 cell inflammatory responses by LPCAT2 inhibition was not restricted to stimulation with TLR4 ligands. RAW264.7 cells respond strongly to stimulation with lipoteichoic acid (LTA) or Pam_3_Cys, which are TLR2/6 and TLR2/1 ligands respectively^26^. We found that cytokine gene expression was also markedly suppressed in cells with siRNA knockdown of LPCAT2 stimulated with either LTA or Pam_3_Cys (data not shown). However, the inhibition of LPCAT1 by siRNA had no effect on either LTA or PAM_3_Cys-induced responses (data not shown).

**Figure 4:**
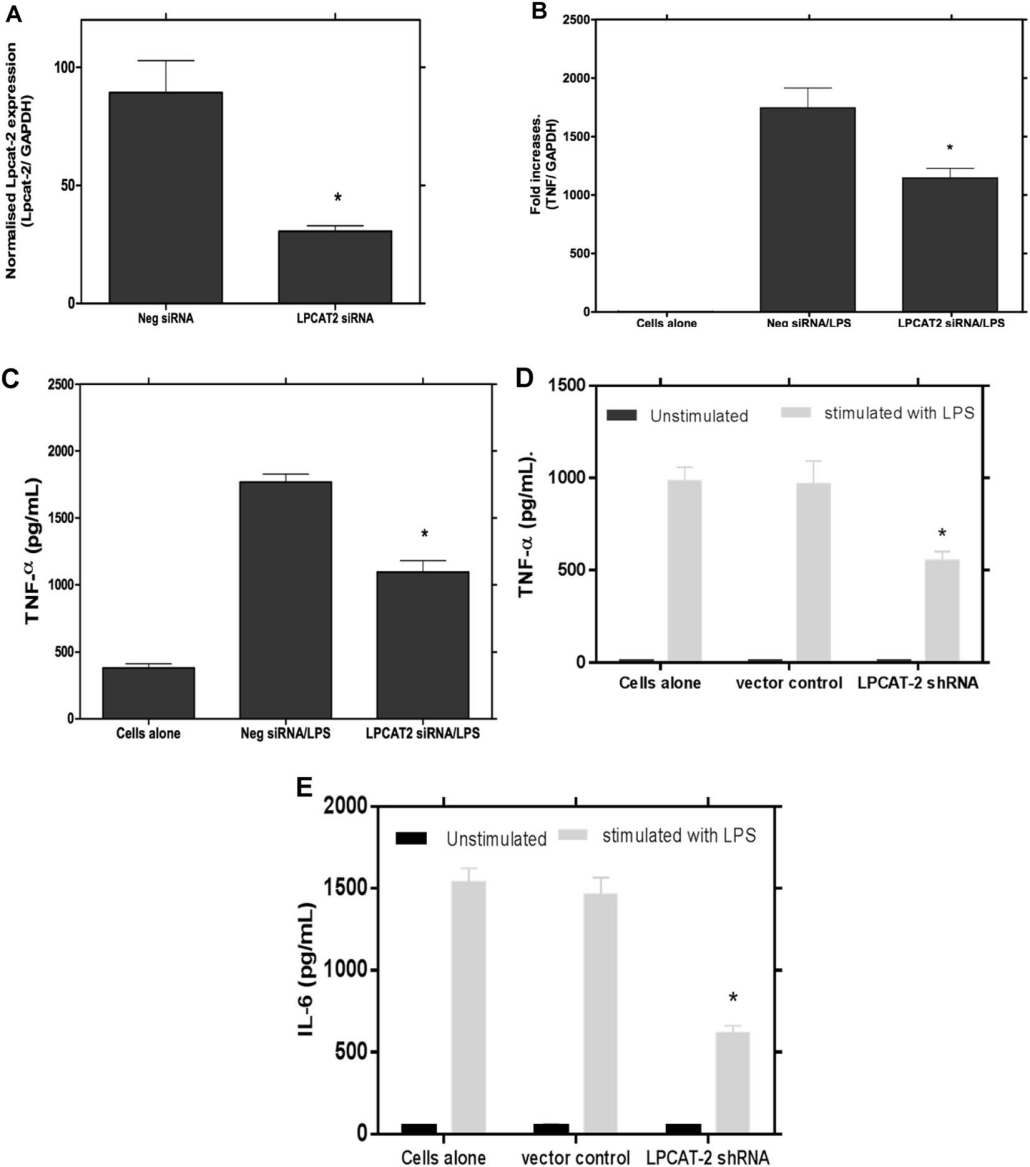
LPCAT2 silencing reduces LPS-induced TNF-α gene expression and protein release from mouse peritoneal macrophages and a human monocyte cell line. LPCAT2 expression was significantly reduced by incubation of primary mouse peritoneal macrophages with LPCAT2 siRNA (p = 0.0192 vs control negative siRNA) (**A**). LPCAT2 silenced primary macrophages show a significant reduction in TNF-α gene expression (**B**) and protein release (**C**) following stimulation with LPS (100ug/ml). Transduction of the human monocytic cell line, MM6, with shRNA lentiviral particles targeting the LPCAT2 gene resulted in reduced production of TNF-α (**D**) and IL-6 (**E**) cytokines following LPS stimulation. *p < 0.05 vs negative siRNA (**B,C**) vs vector control (**D,E**).

To confirm these results in *ex vivo* cells, LPCAT1 and 2 were knocked down by siRNA in peritoneal macrophages which were collected and isolated as described by Zhang *et al*., 2008^23^. The expression level of LPS-induced TNFα mRNA and protein release from peritoneal macrophages stimulated with LPS was significantly reduced when the expression of the LPCAT2 was suppressed (Fig. 4A–C). However, as with the RAW264.7 cells, siRNA knockdown of LPCAT1 had no effect on LPS-induced inflammatory cytokine gene expression in the peritoneal macrophages (results not shown).

The effect of LPCAT2 on LPS-induced responses was not unique to murine macrophages. When the expression of LPCAT2 was silenced in the human monocyte cell line MonoMac6 (MM6) using shRNA, there was also a marked down-regulation of pro-inflammatory cytokines when the cells were stimulated with LPS (Fig. 4D,E).

### Overexpression of LPCAT2 produces enhanced inflammatory responses

**Figure 5:**
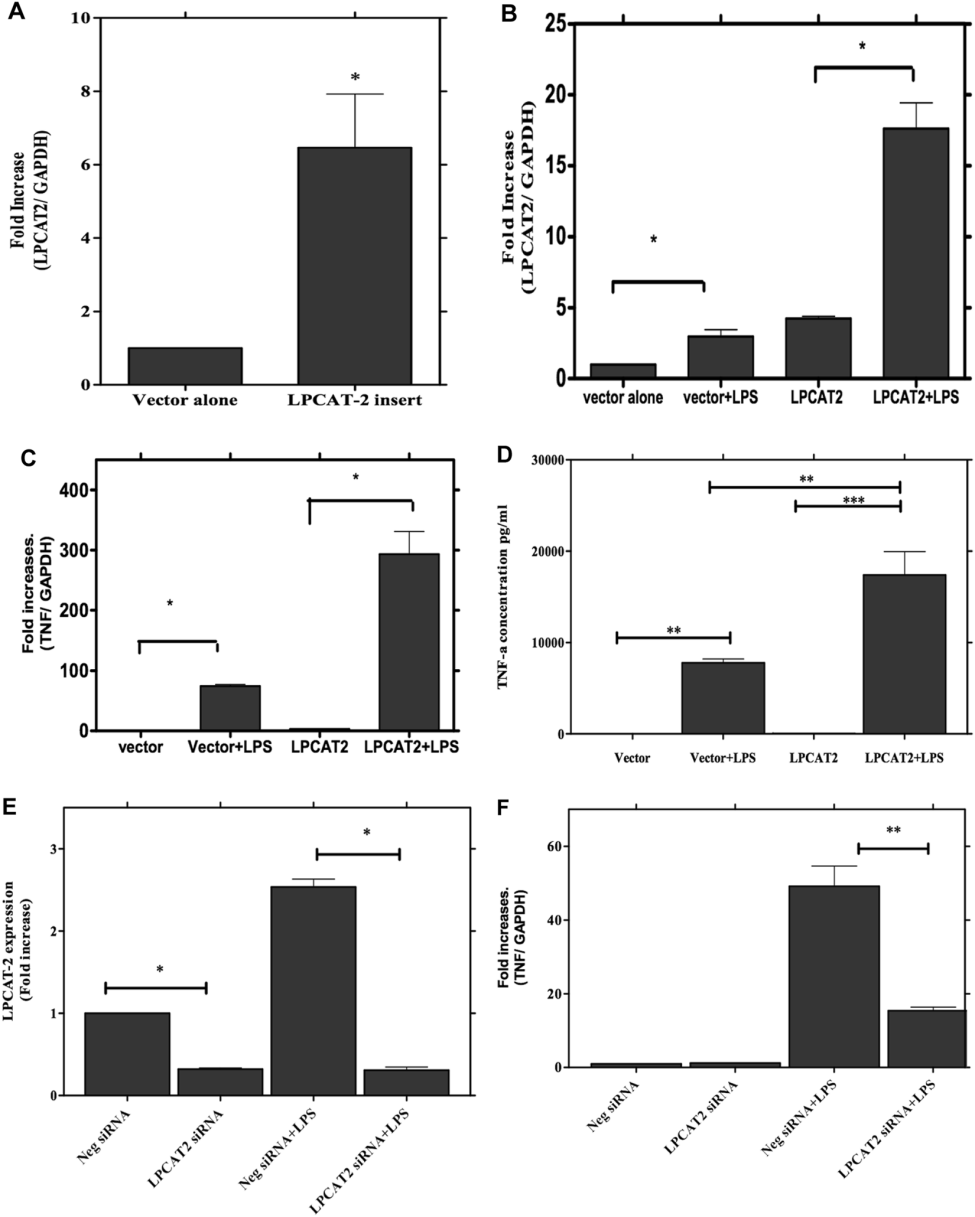
Overexpression of LPCAT2 gene markedly upregulates LPS-induced TNFα gene expression and protein release. The RAW264 cells were transfected with plasmid carrying the LPCAT2 insert (labelled as LPCAT2), which results in a significant increase in the LPCAT2 gene expression (**A**). This overexpression is further increased when the cells were stimulated with LPS (**B**). The overexpression of LPCAT2 significantly increases TNFα gene expression (**C**) and protein release (**D**). siRNA silencing significantly reduced LPCAT2 expression in RAW264 cells carrying the LPCAT2 plasmid (**E**) and significantly inhibited TNFα gene expression in these cells **(F)**. Data represents the mean of four independent experiments (n = 4) ± standard error. *p < *0.05*, **p < *0.01*, **p < *0.001*.

We have shown above that selective inhibition of LPCAT2 gene expression inhibits the inflammatory response to the TLR4 and TLR2 ligands, LPS and LTA. To further show the role of LPCAT2 in macrophage responses to bacterial ligands, RAW264.7 cells were transfected with a plasmid carrying the LPCAT2 gene insert. Macrophages carrying the insert showed increased expression of LPCAT2 compared with cells transfected with empty vector (Fig. 5A). Expression of LPCAT2 was further enhanced by LPS stimulation (Fig. 5B) confirming the induction of the transfected LPCAT2. Cells overexpressing LPCAT2 showed increased expression of TNF-α gene (Fig. 5C) and protein (Fig. 5D) following stimulation with LPS. Moreover, when the cells transfected with LPCAT2 were also subject to siRNA knockdown of LPCAT2 (Fig. 5E), the LPS stimulated gene expression of TNF-α was again significantly inhibited (Fig. 5F). These results support a key role for LPCAT2 in macrophage inflammatory responses.

### LPCAT2 does not mediate cell responses to TLR-independent ligands

**Figure 6:**
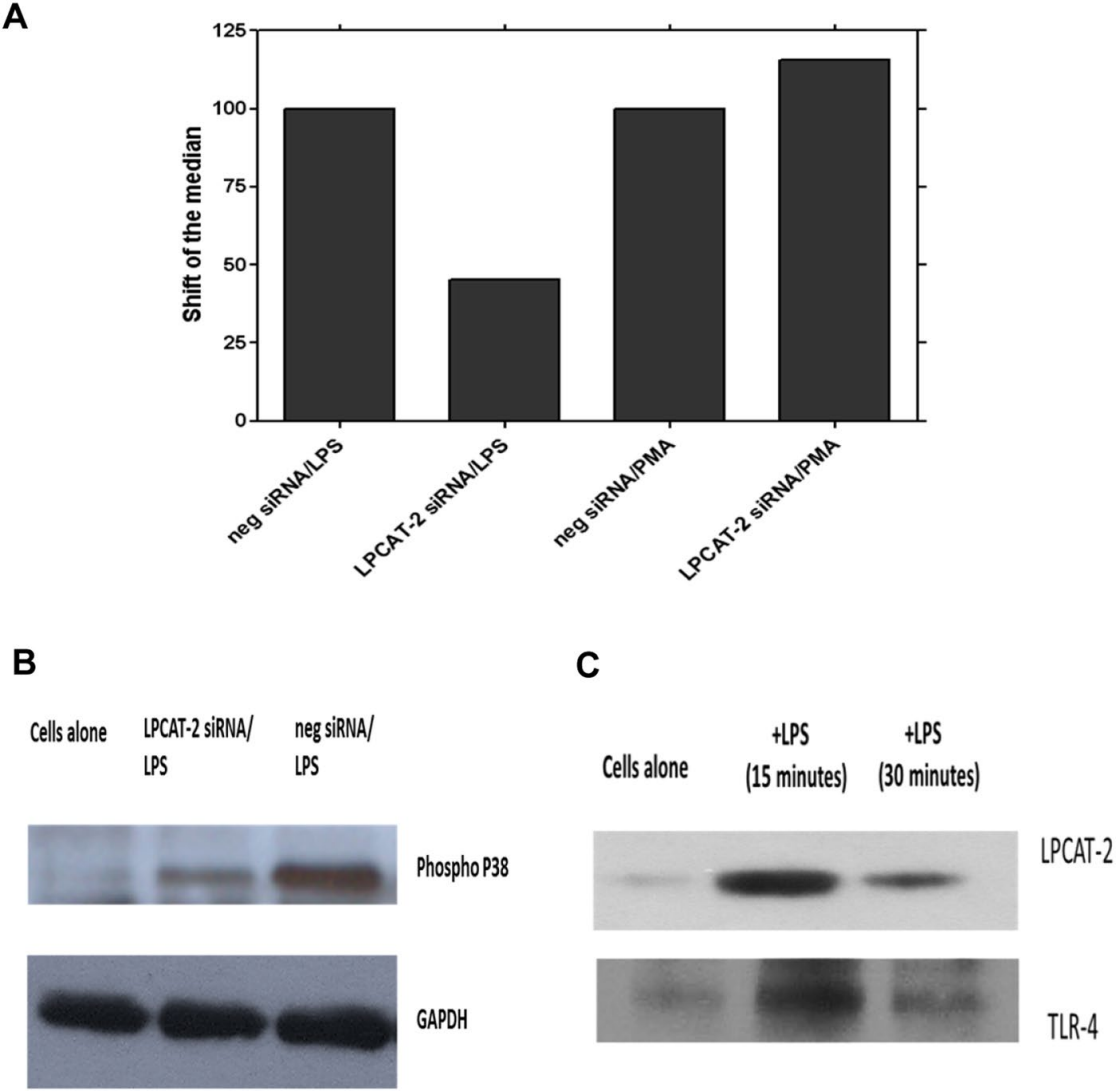
LPCAT2 inhibition had no effect on PMA-induced total reactive oxygen species (ROS) generation while it suppressed LPS-induced ROS production (**A**). When RAW264.7 cells were stimulated with LPS 30 minutes after they had been transfected with LPCAT2 or control siRNA for 24 hours, LPS-induced activation of p38 was significantly reduced in cells treated with siRNA to LPCAT2 (**B**). LPS stimulation also induces the physical association between LPCAT2 and TLR4 (**C**). The physical association of LPCAT2 and TLR4 was demonstrated by immuno-precipitation (IP) after the cells were stimulated with LPS for 15 or 30 minutes. LPCAT2 and TLR4 were detected by immunoblotting following IP using anti-TLR4 and anti-LPCAT2 antibodies respectively (**C**).

To assess the role of LPCAT2 on cell responses to TLR-independent ligands, LPCAT2 expression was silenced in RAW264.7 cells that were stimulated with the soluble activator phorbol 12-myristate 13-acetate (PMA) and cell activation was determined by reactive oxygen species (ROS) generation using flow cytometry. As shown in Fig. 6A, there was no difference in ROS production in response to PMA when cells were transfected with control negative siRNA or LPCAT2 siRNA. However, ROS generation was significantly impaired in LPCAT2 siRNA transfected cells stimulated with LPS. This result indicates that LPCAT2 mediates cell inflammatory responses to TLR ligands but not to TLR-independent stimuli.

### LPCAT2 does not affect CD14, TLR2 or TLR4 Expression

To investigate how LPCAT2 might regulate macrophage inflammatory responses to bacterial ligands, the expression of the proximal signalling receptors CD14, TLR2 and TLR4 was determined in LPCAT2 siRNA transfected and control cells after stimulation with LPS or LTA for 4 hours. Inhibition of LPCAT2 had no effect on the expression of CD14, TLR4 or TLR2 mRNA expression after cell stimulation with either LPS or LTA. LPCAT2 silencing also had no effect on the expression of cell surface TLR2 or TLR4 (data not shown).

### LPCAT2 is required for LPS-induced p38 phosphorylation

The mitogen-activated protein kinases (MAPKs) are crucial serine/ threonine signalling kinases which are activated by phosphorylation resulting in regulation of cellular responses through the modulation of gene expression in the nucleus^27^. LPS has been shown to induce phosphorylation of p38 and activated p38 regulates LPS-induced inflammatory responses^28–30^. Therefore we assessed the impact of LPCAT2 inhibition on p38 phosphorylation following cell stimulation with LPS. As expected, there was a significant up-regulation of p38 phosphorylation after 30 minutes of LPS treatment in control cells transfected with negative siRNA. However, the phosphorylation of p38 was markedly reduced in cells in which LPCAT2 was silenced before stimulation with LPS (Fig. 6B). This suggests that LPCAT2 is required for complete phosphorylation of p38 following cell stimulation with LPS.

### LPS stimulation induces association between TLR4 and LPCAT2

The physical association of LPCAT2 with TLR4 in resting cells and cells stimulated with LPS was examined to further clarify the role of LPCAT2 in LPS-induced responses. The cell lysate was pre-cleared with normal antibodies raised in the same species that anti-TLR4 and anti-LPCAT2 antibodies were raised to reduce non-specific binding before immune-precipitation (IP) was carried out using anti-TLR4 or anti-LPCAT2 antibodies. In unstimulated cells, there was minimal association between TLR4 and LPCAT2. However, following stimulation with LPS for 15 minutes, immunoprecipitation of TLR4 revealed LPCAT2 to be associated with it. This association was transient and had reduced by 30 minutes (Fig. 6C). Similarly, the association between TLR4 and LPCAT2 was also seen in LPS-stimulated cells when LPCAT2 was immune-precipitated and TLR4 was probed (Fig. 6C). When the association between LPCAT1 and TLR4 was explored in a similar fashion, no association of these two proteins was found following stimulation of the cells with LPS (data not shown).

Taken together, these results show that following LPS stimulation, inducible LPCAT2 translocates to membrane lipid raft domains and directly interacts with TLR4 to regulate inflammatory responses in macrophages. This demonstrates a novel role for a phospholipid metabolising enzyme in macrophage activation and inflammatory responses.

## Discussion

Macrophages play a key role in a variety of inflammatory conditions including sepsis, a life-threatening organ dysfunction caused by a dysregulated host response to infection which has proved extremely difficult to treat and carries a high mortality^1,31,32^. We previously reported that LPCAT is important for macrophage inflammatory responses to LPS and other bacterial stimuli, although the isoform of LPCAT responsible for these effects was unknown^11,21,22^. Using the LPCAT sequence data and RNAi technology, we now show that LPCAT2 plays a key role in the inflammatory responses of macrophages to stimulation with bacterial ligands. We demonstrate that in contrast to LPCAT1, LPCAT2 is required for cytokine gene expression in response to TLR4 and TLR2 ligand stimulation. Furthermore, our results indicate that this activity may be elicited through direct association of LPCAT2 with TLR4 in membrane lipid raft domains.

### LPS induces LPCAT2 translocation to membrane lipid raft domains and association with TLR4

The tissue locations and activities of LPCAT1 and LPCAT2 are the subject of current debate. LPCAT1 has been found to be highly expressed in lung tissue where it regulates surfactant lipid generation^17,18^. LPCAT2 has been reported to be expressed in inflammatory cells and to be up-regulated by LPS stimulation^15^. However, previous studies have not reported on the expression level of these enzymes simultaneously within a cell type. Here, we determined the exact copy number of endogenous LPCAT1 and LPCAT2 in RAW264.7 macrophages. Surprisingly, under resting conditions, LPCAT1 was more abundantly expressed than LPCAT2 in these cells. However, after 6 hours of stimulation with the bacterial ligands LPS or LTA, the expression of LPCAT2 was dramatically and significantly increased and remained elevated for over 24 hours of stimulation while the level of LPCAT1 remained unchanged. Such induction of LPCAT2 expression has been reported previously in thioglycolate-elicited murine macrophages although it required 16 hours of LPS stimulation^15^. Our immunoprecipitation experiments revealed that LPS stimulation caused a rapid and reversible association between LPCAT2 and TLR4 (Fig. 6C). Such an association was not evident with LPCAT1. This result, together with the inhibition in macrophage responses following knockdown of LPCAT2 expression, strongly suggests that the association between LPCAT2 and TLR4 facilitates activation of the TLR4 signalling pathways and gene expression.

We further examined the sub-cellular localisation of LPCAT. Both LPCAT1 and LPCAT2 were expressed in the membrane and nuclear fractions but not in the cytosolic fraction of the RAW264.7 cells. Interestingly, LPS stimulation of the cells did not increase the amount of LPCAT1 or LPCAT2 in the nuclear fraction. This is in contrast to a recent study that reported the cytosolic presence of LPCAT1 in a murine lung epithelial cell line and its translocation to the nucleus by LPS stimulation^33,34^. The difference in cytosolic expression could be due to differences in the cell types (lung epithelial versus macrophage) and in the methodology used. In our experiments, following separation of the nuclei, the cell lysate was further spun to separate the cytosolic and membrane fractions. However the studies by Zou and co-workers^33,34^ considered the whole lysate as cytosolic following isolation of the nuclei. These authors further showed that LPCAT1 can acylate histone and modulate gene expression^33^. The biological significance of the presence of LPCAT2 in the nuclei of macrophages is currently under investigation in our laboratory but it is possible that LPCAT2 may also acylate or acetylate nuclear proteins and regulate gene expression in macrophages.

In resting cells, we found LPCAT1 and LPCAT2 to be located mainly in the non-lipid raft fractions of the cell membranes. A previous study reported that LPCAT2 exists both in the raft and non-raft domains of an intestinal epithelial cell line^35^. However, our study shows that following stimulation with LPS, a significant portion of the LPCAT2 translocates into the lipid raft domains while LPCAT1 remains only in the non-raft fraction. This demonstration that LPCAT2 can translocate to raft domains in activated cells, coupled with our findings of the LPS-induced association between LPCAT2 and TLR4, suggest an important step in the mechanism by which this enzyme can regulate inflammatory pathways in macrophages.

### LPCAT2 mediates the inflammatory response to LPS

This study has shown that LPS induces the translocation of LPCAT2 into membrane raft domains and stimulates the association between LPCAT2 and TLR4. Thus, we hypothesise that through these mechanisms LPCAT2 participates in the regulation of inflammatory responses to LPS. We previously showed, using a pharmacological inhibitor, that LPCAT could regulate inflammatory responses to LPS^11,21,36^. However, the LPCAT isoform responsible for this regulation and potential mechanisms were unknown. Here we show, using siRNA knockdown experiments, that LPCAT2 is the isoform that mediates inflammatory responses to LPS and other bacterial ligands. The inhibition of inflammatory gene expression by LPCAT2 knockdown was significant but not total. This may reflect the incomplete inhibition of LPCAT2 mRNA expression by siRNA used (80%) and that other molecules might also regulate these responses. However, the major reduction in cytokine gene expression in response to LPS stimulation by LPCAT2 knockdown suggests that this enzyme plays a significant role in controlling these inflammatory responses. In addition, knockdown of LPCAT2 resulted in inhibition of LPS-induced phosphorylation of the critical p38 MAPK suggesting that LPCAT2 acts upstream of p38. In contrast, LPCAT1 was found to have no role in the induction of inflammatory responses in macrophages. Moreover, LPCAT2 was found to specifically regulate TLR-mediated responses, as ROS production in response to the TLR-independent soluble stimulus PMA was not inhibited in the siRNA transfected RAW cells while LPS-induced ROS production was inhibited. The regulation of TLR-mediated responses by LPCAT2 was not restricted to the RAW264.7 macrophages. Knockdown of LPCAT2 in murine peritoneal macrophages also resulted in significant inhibition of LPS stimulated inflammatory cytokine expression and protein release. This suggests that the LPCAT2 mediated regulation of macrophage inflammatory responses is not restricted to cell lines but also occurs in cells *in vivo*. Furthermore, the activity of LPCAT2 in regulating inflammatory responses was not restricted to murine cells. Knockdown of LPCAT2 expression using shRNA in the human monocyte cell line, MM6, also significantly inhibited the inflammatory cytokine responses to LPS. This suggests that LPCAT2 regulation of inflammatory gene expression to bacterial stimulation may be a common mechanism in innate immune cells. Interestingly, this regulation was not mediated by affecting the transcript level or surface expression of the Toll-like receptors or the accessory molecule CD14.

The acyltransferase activity of LPCAT2 plays an important role in the storage of phospholipid precursors of PAF and eicosanoids^37^. LPCAT2 catalyses the membrane biogenesis (LPCAT activity) of inflammatory cells while producing PAF (LPAFAT activity) in response to external stimuli^15^. However, this is the first report of LPCAT2 mediating inflammatory protein expression in innate immune cells. Since the level of LPCAT2 in the membrane raft fraction increased following LPS stimulation and we found a physical association between TLR4 and LPCAT2, we hypothesise that LPCAT2 may facilitate the recruitment of signalling proteins or their adaptors to lipid raft domains thereby affecting the TLR-induced responses. A recent report has demonstrated that LPS induces phosphorylation of LPCAT2 by MAPK-activated protein kinase 2 (MK2) to affect enzyme activity^38^. We do not know if phosphorylation of LPCAT2 is also required to mediate its effect on inflammatory gene expression. Previously, we showed that pharmacological inhibition of LPCAT activity also inhibited inflammatory gene expression in response to LPS^11,21,36^. This suggests that the acyltransferase activity of LPCAT2 might also be required for modulating the cytokine gene expression seen in this study. As LPCAT2 has been shown to have both LPCAT and LPAFAT activity^15^ it remains to be determined which activity may have effects on TLR-induced gene expression.

In conclusion, our results show that in response to cell stimulation with LPS, LPCAT2, a member of the lysophospholipid acyltransferases, translocates to membrane lipid raft domains, and associates with TLR4 to facilitate inflammatory gene expression via activation of p38 MAPK. This study has shown that an enzyme that regulates membrane phospholipid metabolism can also regulate innate immune inflammatory protein expression in response to bacterial ligands. LPCAT2 may thus be a novel regulator of inflammation in innate immune cells and highlights LPCAT2 as a potential novel target in the development of new therapies for conditions with unregulated inflammatory responses such as sepsis.

## Materials and Methods

Most of the reagents were obtained from Sigma-Aldrich® UK unless otherwise stated. The siRNAs, reverse transcription and Power SYBR Green® kits, OPTI-MEM medium, Geneticin antibiotic, Lipofectamine2000 transfection reagent and pre-cast SDS-PAGE gels were from Life Technologies, UK. INTERFERin was bought from PolyPlus (supplied by Source Bioscience, UK). PE-conjugated Anti-TLR-2 and -4 were supplied by eBioscience Ltd (UK) while antibodies for phospho p38, histone H3, and β-tubulin were supplied by New England Biolab (UK). Anti-LPCAT2 antibodies were bought from Santa Cruz Biotechnology Inc. (supplied by Insight biotechnology, UK) and Novus Biologicals (UK) while LPCAT1 antibody is from Proteintech Europe (UK). Anti-TLR4 and anti-Giα2 antibodies and Protein A/G Plus agarose Bead were from Santa Cruz Biotechnology Inc. (supplied by Insight biotechnology, UK). Anti-GAPDH antibody, PVDF membrane, Milliplex multiple analyte panel (MAP) mouse multiplex kit and PAM3Cys were from Merck-Millipore UK Ltd (UK). The True ORF Gold plasmids with or without murine LPCAT- 2 insert, anti-FLAG antibody and Alpha-Gold competent bacteria were supplied by Origene, USA. All cell culture consumables were from Lonza Ltd (UK) except the foetal bovine serum which was obtained from BioSera (supplied by Labtech International Ltd, UK). Total reactive oxygen species (ROS) detection kit was supplied by ENZO Life Sciences (UK) while the human IL-6 and TNF-α ELISA DuoSet kits were purchased from R&D systems Europe Ltd UK.

### Cell lines and culture

The RAW264.7 murine macrophage cell line was from ECCAC (supplied by Culture Collection, Public Health England, UK). The human monocyte cell line MonoMac 6 (MM6) was obtained from the German collection of microorganisms and cell cultures (DSMZ, Germany). RAW264.7 cells were cultured in DMEM Medium supplemented with 10% foetal bovine serum (FBS) and 2 mM glutamine (DMEM complete medium) at 37 °C in a 5% CO_2_-humidified atmosphere. MM6 cells were cultured in RPMI-1640 medium, supplemented with 10% FBS, 1% 2 mM L-glutamine, 1% nonessential amino acids and 1% sodium pyruvate at 37 °C in a 5% CO_2_-humidified atmosphere.

### Animals

The peritoneal macrophages were harvested from C57BL/6 mouse strain. The mice were bred locally and were used between 6 to 8 weeks old. All experimental protocols were approved by Plymouth University regulations under UK Home Office license and all methods were carried out in accordance with relevant guidelines and regulations. All mice were maintained at the Plymouth University Animal Care Facility. The animals were fed a standard chow pellet diet and had free access to water. All animals were maintained on a 12-h light, 12-h dark cycle. The resident peritoneal macrophages were collected and isolated as described by Zhang *et al*., (2008)^23^ following CO_2_ euthanasia.

### Expansion and purification of plasmids

The True ORF Gold plasmids which carries a kanamycin resistance gene (with and without murine LPCAT2 insert) was expanded using Alpha-Gold competent bacteria according the supplier instruction. Briefly; 1 µg/mL of the plasmid are incubated with 10 µL of the bacteria for 30 minutes on ice followed by incubation at 42 °C for 45 seconds. The transformed bacteria colonies were selected by growing the bacteria on a LB-plate containing 25 µg/ml of Kanamycin. The colonies was transferred into an LB broth containing kanamycin at a concentration of 60 µg/mL and incubated while shaken for 12 hours. The plasmids are purified using GenEluteTM Plasmid Miniprep Kit according the supplier instruction.

### Transfection RAW264 cells with plasmid containing the LPCAT2 insert

RAW264.7 cells at a concentration of 10^6^ cells/well were seeded on a 6-wells plate (2.5 mL of DMEM medium) and incubated overnight. The cells were then transfected with a vector or plasmid containing the LPCAT2 insert (2.5 µg) in the presence of 9 µL Lipofectamine2000 TM using OPTI-MEM medium. After 24 hours, the full DMEM medium is changed and the cells further incubated for 24 hours. The over-expression was checked by RT-PCR and western blotting.

### siRNA and shRNA inhibition of LPCATs

RAW264.7 cells at 2 × 10^5^cells/2.5 mL/well (for RT-PCR) or 7 × 10^4^ cells/1 mL/ well (for multiplex cytokine protein assay) were seeded onto 6-well or 12-well plates respectively in DMEM complete medium and incubated for 24 h. The primary peritoneal macrophages once isolated seeded at 0.25 × 10^6^cells/mL onto 25 cm^2^ flasks (4 mL) in DMEM complete medium containing penicillin/ streptomycin and antimycotic agent and incubated for 24 h. The cells were then transfected in serum free OPTI-MEM medium with a control negative siRNA (Ambion), or siRNA directed against LPCAT1 (Ambion siRNA ID s102346) or LPCAT2 (Ambion siRNA ID s114512) at a final concentration of 5 nM (for RAW264.7) and 10 nM (peritoneal macrophages) using INTERFERin transfection reagent. After 24 h, the medium was changed and complete DMEM medium added and the cells were stimulated with the bacterial agonists. For the shRNA experiments, the lentiviral particles produced from sequence verified lentiviral plasmid vectors pLK0.1-TRC-shRNA -puro, targeting the LPCAT2 gene, were purchased from Sigma Aldrich UK (Mission® TRC shRNA TRCN0000055780). As a control, shRNA deficient pLK0.1-TRC- puro lentiviral particles were used. MM6 cells were seeded at a density of 1.6 ×10^4^ per well of a 96 well plate and transduced with pLK0.1-TRC-shRNA –puro expressing lentivirus or pLK0.1-TRC-puro control lentivirus at a multiplicity of infection (MOI) of 50 in the presence of hexadimethrine bromide. Cells were cultured for 72 hours, with regular medium changes before puromycin selection (0.5 µg/ml) of shRNA expressing cells.

### Reverse transcription and real time quantitative PCR (RT-qPCR)

Total RNA was extracted using guanidinium thiocyanate-phenol-bromochloropropane and the total RNA concentration was determined using NanoVue spectrophotometer (GE Healthcare, UK). The purity was assessed by examining the ratio of the absorbance measurement at 260 to 280 nm while the integrity was assessed by running a gel electrophoresis on 1% denaturing agarose gel. The reverse transcription was done using High Capacity RNA-to-DNA kit for 0.5 µg total RNA according to manufacturer’s instructions. The quantitative real time PCR was performed using Power SYBR Green and StepOnePlus thermal cycler (Applied Biosystems, UK). The primers for LPCAT1, LPCAT2, TNF-α, IL-6, IL-10, GAPDH, CD14, TLR2 and TLR4 (Table 1) were designed using Primer Express 3.0 software (Applied Biosystems, UK) to amplify 100 bp product for all the targets.

**Table 1:** Sequences of primers for real time quantitative PCR.

Target	Primer sequence (5’→3’)	Accession number
LPCAT1	Forward: TGGCTCACTCTGTGCCAGTT	NM_145376.5
	Reverse: TGGCATACAGGGCAGGATTC	
LPCAT2	Forward: GCACACGCTCCCCTTGACT	NM_173014.1
	Reverse: TTATGGCCCACTGCAATCGT	
IL-6	Forward: AGAAGGAGTGGCTAAGGACCAA	NM_031168.1
	Reverse: ACGCACTAGGTTTGCCGAGTA	
IL-10	Forward: CTGGCTCAGCACTGCTATGCT	NM_010548.2
	Reverse: ACTGGGAAGTGGGTGCAGTT	
TNF-α	Forward: AGGACCCAGTGTGGGAAGCT	NM_013693.2
	Reverse: AAAGAGGAGGCAACAAGGTAGAGA	
GAPDH	Forward: CCTCGTCCCGTAGACAAAATG	NM_008084.2
	Reverse: TCTCCACTTTGCCACTGCAA	
CD14	Forward: GCAGTGGCCTTGTCAGGAA	NM_009841.3
	Reverse: CGTTGACGAGGACCCTCAGA	
TLR2	Forward: AGGGATCCGGGTGGTAAAAA	NM_011905.3
	Reverse: CAGCCGAGGCAAGAACAAAG	
TLR4	Forward: ACTCTGATCATGGCACTGTTCTTCT	NM_021297.2
	Reverse: TCTGATCCATGCATTGGTAGGT	

### Relative quantitation of LPCAT1 and 2 expressions

The copy numbers of LPCAT1 and 2 were determined using a relative standard curve of a known concentration of a 100 bp PCR product (GAPDH). A 7-point standard was prepared using this PCR product by serial dilutions (1:5) in the range of 0.8 ng/µL to 5.12 ×10^−5^ ng/µL. The quantitative real time PCR was performed using primer sets designed to produce 100 bp product for LPCAT1 and 2, on cDNA generated from the reverse transcription of the resting (naive) cells to determine the expression and the exact copy number of the two LPCATs per cell. The amount of LPCAT1 and 2 in ng/µL was calculated using a formula 10 (Ct-b)/m, where Ct is the threshold number, b is y-intercept and m is the slope. The total amount of the PCR product in the total reaction mix was then calculated by multiplying the concentration by the total volume of the reaction mix. Thereafter, the copy number was calculated retrospectively using molecular weights of the mRNAs (LPCAT1 = 33004.2 g/mol and LPCAT2 = 32564 g/mol), the actual mass in the reaction mix, cell density and Avogadro’s number (6.02 × 10^23^).

### Multiplex cytokine assay

Cytokines were measured using a Milliplex MAP mouse multiplex kit according to the manufacturer’s instructions. Calibration curves from the recombinant cytokine standards were prepared by serial dilutions in the same media as the culture supernatant (DMEM medium containing 10% foetal bovine serum). High and low reference points for each cytokine were included to determine cytokine recovery. The level of detection for each cytokine assayed was 3.2–10000 pg/mL.

### Measurement of TNF-a and IL-6 release from human monocytic cells

To determine the differential effects of reduced LPCAT2 gene expression on cytokine production in response to LPS in human monocytic cell line, control and shRNA lentivirus transduced MM6 cells were incubated at a density of 0.5 × 10^6^ /mL in the presence or absence or LPS (100 ng/ml) for 4 or 18 hours. Following incubation, supernatants were collected and stored at –80 °C. TNF-α and IL-6 in cell culture supernatants were quantified by ELISA in accordance with the manufacturer’s instructions.

### Lipid raft isolation by successive detergent extraction

The lipid raft, non-lipid raft fractions, the cytosolic and nuclear fractions were separated using successive detergent extractions as described previously^24^ with minor modification. Briefly, once the experiment was finished, cells were washed twice with ice-cold PBS, scraped off and pelleted at 160 × g for 5 minutes. The cells were re-suspended in ice-cold Tris-buffer (50 mM, pH = 7.5) containing 150 mM NaCl and protease inhibitor and homogenised using the HGM Cell Cracker (HGM, Heidelberg, Germany). The lysate was spun at 500 × g for 5 minutes to pellet the nuclei and the supernatant was further centrifuged at 20,000 × g for 20 minutes to separate the cytosolic and the membrane fractions. The non-raft fraction was isolated by re-suspending and incubating the membrane fraction in 2-(N-morpholino)-ethanesulphonic acid (MES)-buffered saline (MBS) (25 mM MES (pH = 6.5) containing 150 mM NaCl, 1% Triton X-100 and protease Inhibitor) for an hour on ice. The lipid raft fraction was isolated by treating the Triton-insoluble fraction that was recovered after the centrifugation at 20,000 using 60 mM a Tris-buffer containing β-octylglucoside.

### Immunoblotting

Cell membrane fractions with equal amounts of protein were separated on NuPAGE® pre-cast SDS-PAGE and blotted on to a PVDF membrane using a blot module (Life Technologies, UK). The blots were blocked with 0.1% low fat dried skimmed milk powder in PBS-0.1% Tween 20®, probed with primary and then HRP-conjugated secondary antibodies using SNAP i.d. TM protein detection system (Millipore, UK). The proteins on the membrane were detected using a Luminata Crescendo Western HRP substrate (Millipore, UK) and analysed using ImageJ.

### Flow cytometry

Flow cytometry analyses were performed using a FACSAria III flow cytometer (BD Biosciences, USA). The RAW264.7 cells were gated and the cell debris were discounted using the forward and side scatters profile and 10,000 events were acquired using BD FACSDivaTM software (BD Biosciences, USA) and analysed using WinMDI 2.8 software [Joe Trotter, Pharmingen, CA, USA].

### Oxidative burst

Total reactive oxygen species (ROS) production was measured in real time by flow cytometry with a Total ROS detection kit as per the supplier’s instructions. Briefly, 0.5×10^6^ cells were seeded onto T25 culture flasks in complete DMEM medium. After 24 h incubation, the cells were transfected with either negative or LPCAT2 siRNA using OPTI-MEM medium. The cells from each flask were scraped off and stimulated with 1 µg/mL LPS or 1 µM phorbol 12-myristate 13-acetate (PMA). After 20 minutes incubation, the cell suspensions were spun and the cell pellets were washed with the wash buffer supplied with the kit. The cells were then re-suspended in ROS detection solution and incubated for 30 minutes at 37 °C in the dark and analysed by flow cytometry. The median fluorescence of the stimulated cells transfected with LPCAT2 siRNA was compared with the cells transfected with negative siRNA as described by Namgaladze *et al*., 2009^25^.

### Data analysis

Data are shown as means + SE from at least three independents experiments performed in duplicate. GraphPad prism (GraphPad Software, La Jolla, California, USA) was used for statistical analysis. Data were compared using ANOVA or student t-test and a p-value of <0.05 was considered statistically significant.
